# Supplementary material for: Deleting a UBE3A substrate rescues impaired hippocampal physiology and learning in Angelman syndrome mice
Source: Sci Rep. 2021 Sep 30;11:19414. doi: 10.1038/s41598-021-97898-w (PMC8484563; doi:10.1038/s41598-021-97898-w)
Supplement: Supplementary file 1 — Supplementary Information. [file 41598_2021_97898_MOESM1_ESM.docx]

**Supplementary Fig. 1. Ephexin5 is ubiquitylated by UBE3A in vitro, has elevated expression in P30 AS hippocampus, and does not contribute to passive avoidance phenotypes, related to Figure 1. (A)** *In vitro* ubiquitylation assays were performed using purified Ephexin5 protein, with different purified components (UBE3A, ATP, E6, or Ephexin5) of the *in vitro* UBE3A ubiquitylation assay as indicated. The arrows indicate the unmodified protein and the bracket indicates the smearing due to ubiquitylation. Samples were prepared and applied to SDS-PAGE for immunoblot analysis using indicated antibodies. **(B)** Ubiquitylated lysines (red K) identified by tandem mass spectrometry analysis of tryptic peptides from Ephexin 5 after *in vitro* UBE3A ubiquitylation. Green arrows indicate identified sites using reactions in the absence of the enhancing protein E6. **(C)** Diagram of sites of ubiquitylation and domains of Ephexin5. Black circles indicate a site of ubiquitylation at the specified lysine residue. DH = Dbl-homology (Rho-GEF) domain, PH = Pleckstrin homology domain. **(D)** Latency for 129S7 mice to enter shock arena on day 1 and day 2. Data are presented as mean ± SEM. **p*<0.05 (two-way ANOVA) compared to Day 1 within genotype with *post-hoc* Bonferroni multiple comparisons test. **(E)** Ephexin5 expression levels are elevated in AS mouse hippocampus at P30 and removed in the AS/E5^-/-^. Quantification of Ephexin5 signal is normalized to Actin signal and compared to WT. Arrow head indicates Ephexin5 band. Data are presented as mean ± SEM (n = 6 for WT, n = 7 for AS). **(F)** Latency for C57Bl/6J mice to enter shock arena on day 1 and day 2. Data are presented as mean ± SEM. **p*<0.05 compared to Day 1 within genotype and ^+^*p*<0.05 compared to Day 2 AS and AS/E5^-/-^ (two-way ANOVA) with *post-hoc* Bonferroni multiple comparisons test. Sample size for behavior (n), degrees of freedom, and exact *p* values are reported in **Supplementary Table 2** and **Supplementary Table 3**. Proteomics analysis used Proteome Discoverer (v1.4 - www.thermofisher.com OPTON-30795), Mascot (v2.5.1- www.matrixscience.com), and Scaffold (v4.8.4 - www.Proteome software.com/products/scaffold-5). Behavior analysis used ODlog (v 2.5 www. macropodsoftware.com/odlog/).


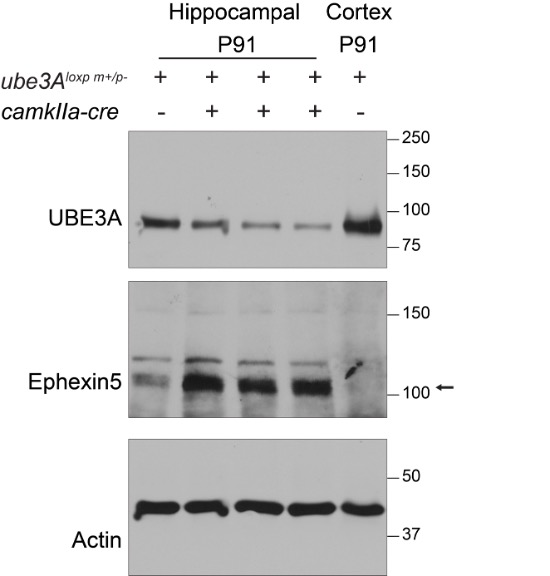


**Supplementary Fig. 2. Ephexin5 expression levels are elevated in hippocampus from *ube3A^loxp+^/camkIIa-cre^+^* mice and not present in the cortex, related to Figure 2.** Red box indicates lanes shown in Figure 2B. Additional lanes show increased Ephexin5 expression in hippocampi from *ube3A^loxp+^/camkIIa-cre^+^* mice. Note that Ephexin5 expression is undetectable in cortical samples. Compare lane 5 to lane 1.


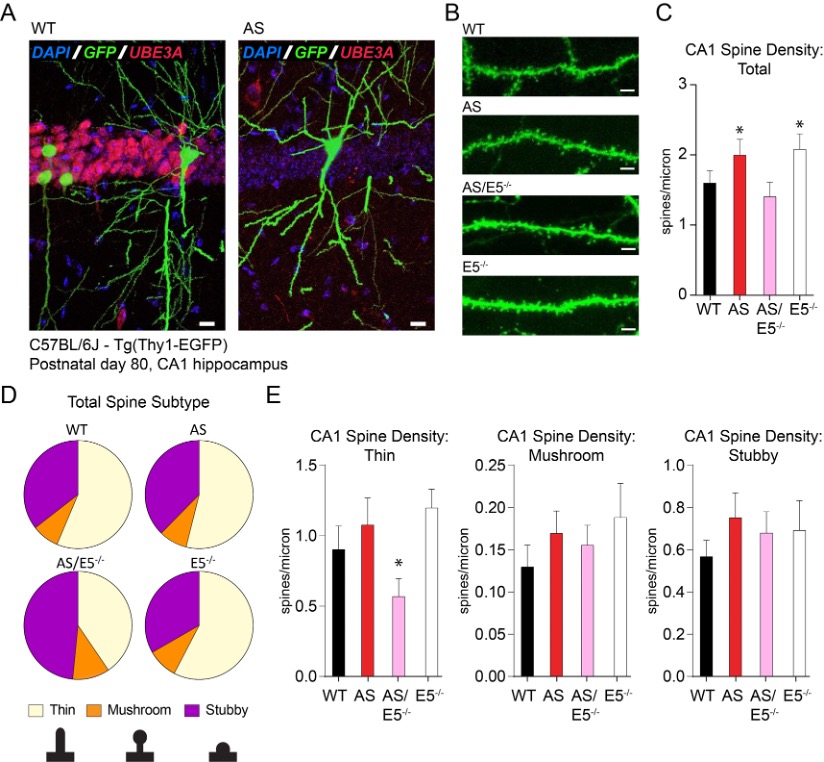


**Supplementary Fig. 3. Spine density is altered in the AS CA1 region, and corrected with removal of Ephexin5. (A)** 11 week old WT and AS Thy1-EGFP-positive tissue was stained for UBE3A (red) and GFP (green), with DAPI (blue) for nuclei labeling. UBE3A is present in CA1 in the WT, but this staining is lost in the AS animal. **(B)** Example dendritic segments from WT, AS, AS/E5^-/-^ and E5^-/-^ CA1. Taken at 63x, scale bar 2 μm. **(C)** Total spine density in CA1. **(D)** Proportion of spine subtypes within WT, AS, AS/E5^-/-^, and E5^-/-^ CA1 neurons. **(E)** Spine density across the CA1 neurons for thin, mushroom, and stubby morphologies. Data are presented as mean ± SEM (n = 3 for all genotypes). **p*<0.05 (one-way ANOVA) compared to WT and AS/E5^-/-^ with *post-hoc* Tukey’s multiple comparisons test. **p*<0.05 (two-way ANOVA) compared to AS and E5^-/-^ with *post-hoc* Sidak’s multiple comparisons test for panel. Degrees of freedom, and exact *p* values are reported in **Supplementary Table 2** and **Supplementary Table 3**. Image analysis was performed using Fiji/ImageJ (v2.0.0 - imagej.nih.gov/ij/docs/guide/146-2.html), and NeuronStudio (research.mssm.edu/cnic/tools-ns.html).


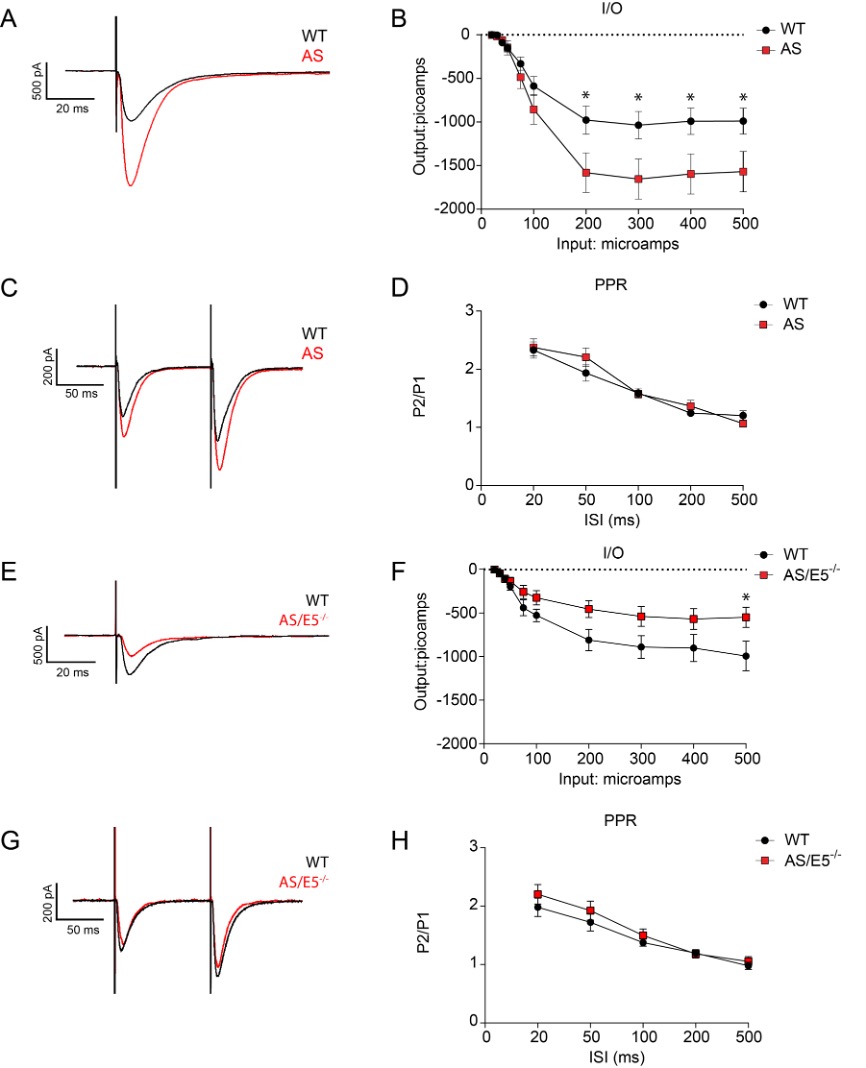


**Supplementary Fig. 4. Electrophysiological properties of CA1 pyramidal neurons in WT, AS, and AS/E5**^-/-^ **hippocampus, related to figure 4. (A)** Example traces showing evoked potentials in the WT and AS CA1 region after stimulation of the Schaffer collaterals. **(B)** Output in picoamps for WT and AS shown for varying input stimulus intensity. Data are presented as mean ± SEM. One-way ANOVA, *post-hoc* Tukey’s multiple comparisons test. Two-way ANOVA, *post-hoc* Bonferroni multiple comparisons test. **p*<0.05 compared to WT. **(C)** Example traces showing the evoked potentials in both WT and AS CA1 region after introduction of two stimuli to determine paired pulse facilitation. **(D)** Ratio of second potential over the first potential is shown for varying interstimulus intervals in WT and AS cells. Data are presented as mean ± SEM. Statistically significant difference between samples was not observed (two-way ANOVA) with *post-hoc* Bonferroni multiple comparisons test. **(E)** Example traces showing evoked potentials in the WT and AS/E5^-/-^ CA1 region after stimulation of the Schaffer collaterals. **(F)** Output in picoamps for WT and AS/E5^-/-^ shown for varying input stimulus intensity. Data are presented as mean ± SEM. One-way ANOVA, *post-hoc* Tukey’s multiple comparisons test. Two-way ANOVA, *post-hoc* Bonferroni multiple comparisons test. **p*<0.05 compared to WT. **(G)** Example traces showing the evoked potentials in both WT and AS/E5^-/-^ CA1 region after introduction of two stimuli to determine paired pulse facilitation. **(H)** Ratio of second potential over the first potential is shown for varying interstimulus intervals in WT and AS/E5^-/-^ cells. Data are presented as mean ± SEM. Statistically significant difference between samples was not observed (two-way ANOVA) with *post-hoc* Bonferroni multiple comparisons test. Sample size (n), degrees of freedom, and exact *p* values are reported in **Supplementary Table 2** and **Supplementary Table 3**. Analysis was done with MiniAnalysis program (v 6.0 - http://www.synaptosoft.com/MiniAnalysis/) and pCLAMP11 (v 10.3.1.4 www.moleculardevices.com).

**Supplementary Table 1: A detailed outline of critical reagents and resources required for this study.**

| REAGENT or RESOURCE | SOURCE | IDENTIFIER |
| --- | --- | --- |
| Antibodies | | |
| Mouse anti-UBE3A (330) | Sigma-Aldrich | Cat# E8655; RRID AB_261956 |
| Mouse anti-UBE3A (3E5) | Sigma-Aldrich | Cat# **SAB1404508; RRID AB_10740376** |
| Mouse anti-β-Actin (8226) | Abcam | Cat# ab8226; RRID AB_306371 |
| Chicken anti-GFP | Aves Labs | Cat#1020; RRID AB_10000240 |
| Rabbit anti-Ephexin5 | M. Greenberg (Harvard) | (Margolis et al., 2010)^12^ |
| Goat anti-Rabbit IgG, (H+L), HRP | Cell Signaling Technology | Cat# 7074S; RRID AB_2099233 |
| Horse anti-Mouse IgG, (H+L), HRP | Cell Signaling Technology | Cat# 7076S; RRID AB_330924 |
| Donkey anti-Mouse Cy3 | Jackson Immunoresearch | Cat# 715-165-151; RRID AB_2315777 |
| Goat anti-Chicken 488 | Thermo Fisher Scientific | Cat# A-11039; RRID AB_142924 |
| Bacterial and Virus Strains | | |
| DH5α | Thermo Fisher Scientific | Cat# 18265017 |
| Rosetta DE3 BL21 bacteria | E. Goley (kind gift) | N/A |
| Chemicals, Peptides, and Recombinant Proteins | | |
| Poly-L-Lysine (PLL) | Sigma-Aldrich | Cat# P4707 |
| Papain | Worthington | Cat# LS003127 |
| B27 | Thermo Fisher Scientific | Cat# 17504044 |
| Neurobasal | Thermo Fisher Scientific | Cat# 21103049 |
| Neurobasal A | Thermo Fisher Scientific | Cat# 10888-022 |
| Penicillin/streptomycin | Sigma-Aldrich | Cat# 15140122 |
| L-Glutamine | Thermo Fisher Scientific | Cat# 25030081 |
| Trypsin | Thermo Fisher Scientific | Cat# 25200056 |
| Trypsin Inhibitor | Sigma-Aldrich | Cat# T9253; CAS: 9035-81-8 |
| Trypsin/LysC (MS/MS) | Promega | Cat# V5071 |
| DMEM | Thermo Fisher Scientific | Cat# 11960069 |
| Fetal bovine serum | Thermo Fisher Scientific | Cat# 16000044 |
| Phosphate buffered saline | Thermo Fisher Scientific | Cat# 10010049 |
| cOmplete Protease inhibitor | Sigma-Aldrich | Cat# 11836170001 |
| Precision Plus Standards | Bio-Rad | Cat# 1610374 |
| PROTOGEL (30%) | Thermo Fisher Scientific | Cat# 50-899-90118 |
| Lipofectamine 2000 | Life Technologies | Cat# 11668019 |
| UBE1 | Boston Biochem | Cat# E-304 |
| E6AP | Boston Biochem | Cat# E3-230 |
| E6 | Boston Biochem | Cat# AP-120 |
| Ubiquitin | Boston Biochem | Cat# U-100H |
| Mg-ATP | Boston Biochem | Cat# B-20 |
| TCEP | Thermo Fisher Scientific | Cat# 20490 |
| Triton-X 100 | Sigma-Aldrich | Cat# T9284 |
| Paraformaldehyde | Thermo Fisher Scientific | Cat# 50980487 |
| Neg50 OCT | Thermo Fisher Scientific | Cat# 6502 |
| Fluoromount-G | Southern Biotech | Cat# 0100-01 |
| Hoechst | Molecular Probes | Cat# H-3570 |
| DNaseI | Qiagen | Cat# 79254 |
| DMSO | Thermo Fisher Scientific | Cat# 25-950-CQC |
| lysozyme | Sigma-Aldrich | Cat# L6876 |
| Clozapine *N*-oxide (CNO) | Tocris | Cat# 4936; CAS: 34233-69-7 |
| TTX | Tocris Bioscience | Cat# 1078; CAS: 4368-28-9 |
| Gabazine | Sigma-Aldrich | Cat# SR-95531; CAS: 104104-50-9 |
| APV | Tocris Bioscience | Cat# S106; CAS: 79055-68-8 |
| 2-mercaptoethanol | Bio-Rad | Cat# 1610710 |
| Nitric Acid | Thermo Fisher Scientific | Cat# A200212 |
| NaCl | Sigma-Aldrich | Cat# S9625 |
| KCl | Sigma-Aldrich | Cat# P9541 |
| IPTG | Sigma-Aldrich | Cat# I5502 |
| Tris base | Sigma-Aldrich | Cat# T1503 |
| PMSF | Sigma-Aldrich | Cat# P7626; CAS: 329-98-6 |
| DTT | Sigma-Aldrich | Cat# D5545 |
| LB broth | Sigma-Aldrich | Cat# L3022 |
| LB plates | Quality Biologicals | Cat# 340-107-231 |
| Critical Commercial Assays | | |
| PureLink HiPure Plasmid Filter Maxiprep Kit | Invitrogen | Cat# K210016 |
| Experimental Models: Organisms/Strains | | |
| C57Bl/6j | Jackson Laboratories | RRID:IMSR_JAX:000664 |
| Ephexin5^-/-^ 129 | M. Greenberg (Harvard) | (Margolis et al., 2010)^12^ |
| Ephexin5^-/-^ C57BL/6 | This study | (Margolis et al., 2010)^12^ |
| 129-*Ube3a^tm1Alb^*/J | Jackson Laboratories | RRID:IMSR_JAX:004477 |
| B6-*Ube3a^tm2Alb^*/J (backcrossed – this study) | Jackson Laboratories | RRID:IMSR_JAX:017765 |
| tdTomato cre reporter lines | Jackson Laboratories | RRID:IMSR_JAX:007909 |
| CamK2a-cre | Jackson Laboratories | RRID:IMSR_JAX:005359 |
| Tg(Thy1-EGFP) MJrs/J | Jackson Laboratories | RRID:IMSR_JAX:007788 |
| Floxed-UBE3A | B. Philpot (kind gift) | (Judson et al., 2016)^25^ |
| Recombinant DNA | | |
| ube3a-shRNA | M. Greenberg (Harvard) | (Greer et al., 2010)^40^ |
| scr-shRNA | M. Greenberg (Harvard) | (Greer et al., 2010)^40^ |
| pcDNA5/FRT-HA-hM4D(Gi) | (Armbruster et al., 2007) ^41^ | Addgene Plasmid # 45548 |
| EGFP | M. Greenberg (Harvard) | (Margolis et al., 2010)^12^ |
| pGEX-GST-Ephexin5 - WT | This Study | N/A |
| Software and Algorithms | | |
| Proteome Discoverer (v1.4) | Thermo Fisher Scientific | Cat# OPTON-30795 |
| Mascot (v2.5.1) | Matrix Science | www.matrixscience.com |
| Scaffold (v4.8.4) | Proteome Software | http://www.proteomesoftware.com/products/scaffold/ |
| Fiji/ImageJ | National Institutes of Health | https://imagej.nih.gov/ij/docs/guide/146-2.html |
| NeuronStudio | (Rodriguez et al., 2008) | http://research.mssm.edu/cnic/tools-ns.html |
| MiniAnalysis program (v 6.0) | Synaptosoft | http://www.synaptosoft.com/MiniAnalysis/ |
| pCLAMP11 (v 10.3.1.4) | Molecular Devices | www.moleculardevices.com |
| ODlog (v 2.5) | Macorpod | www.macropodsoftware.com/odlog/ |
| Other | | |
| 12 mm Glass coverslips | Bellco | Cat# 1943-10012A |
| Glutathione Sepharose | GE Healthcare | Cat # 17513201 |
| Slide-A-Lyzer | Thermo Fisher Scientific | Cat # 66380 |

**Supplementary Table 2: Statistical tests and p values for indicated Figures.**

| **Figure** | **Statistical Test** | **P value** | **Significance** |
| --- | --- | --- | --- |
| 1B – Ephexin5 levels P91 | Student’s T-test | 0.0094 | * |
| 1C - NPP | Two-way ANOVA | Object: p=0.0046  Genotype: p > 0.9999  Interaction: p < 0.0001 | *  ns  * |
| 1D - NOR | Two-way ANOVA | Object: p<0.0001  Genotype: p>0.9999  Interaction: p=0.0620 | *  ns  ns |
| 1E - Rotarod | One-way ANOVA | p < 0.0001 | * |
| 1F - Rotarod | One-way ANOVA | p=0.0006 | * |
| 1G – Marble burying | One-way ANOVA | p=0.0002 | * |
| 2B – Ephexin5 and UBE3A levels in Cre animals | Student’s T-test, Welch’s correction | Ephexin5: 0.0016  UBE3A: 0.0473 | *  * |
| 2C – Rotarod | Student’s T-test | 0.5509 | ns |
| 2D - NPP | One-way ANOVA  Kruskal-Wallis  One-way ANOVA  Kruskal-Wallis | p=0.0002  p=0.2867  p=0.0004  p=0.0039 | *  ns  *  * |
| 3C – Spine density | One-way ANOVA | p < 0.0001 | * |
| 4B – mEPSC frequency | One-way ANOVA | p=0.0008 | * |
| 4C – mEPSC amplitude | One-way ANOVA | p=0.0548 | ns |
| 4E – Rise time | One-way ANOVA | p=0.0016 | * |
| 4F – Decay time | One-way ANOVA | p=0.0287 | * |
| 1-1D – Ephexin5 levels P30 | Student’s T-test | p=0.0722 | ns |
| 1-1E – Passive avoidance | Two-way ANOVA | Genotype: p=0.0016  Day: p < 0.0001  Interaction: p=0.0012 | *  *  * |
| 1-1F – Passive avoidance | Two-way ANOVA | Genotype: p=0.6671  Day: p<0.0001  Interaction: p=0.6671 | ns  *  ns |
| 3-1B – Spine density | One-way ANOVA | p < 0.0001 | * |
| 3-1E – Spine density | One-way ANOVA | p < 0.0001 | * |
| 3-1H – Spine density | One-way ANOVA | p < 0.0001 | * |
| 4-1C – Input/Output | Two-way ANOVA | Stimulus: p < 0.0001  Genotype: p < 0.0001  Interaction: 0.0177 | *  *  * |
| 4-1D - PPF | Two-way ANOVA | ISI: p<0.0001  Genotype:0.4055  Interaction: p=0.4170 | *  ns  ns |
| 4-1F – Input/Output | Two-way ANOVA | Stimulus:p<0.0001  Genotype:p<0.0001  Interaction: p=0.0789 | *  *  ns |
| 4-1H - PPF | Two-way ANOVA | ISI: p<0.0001  Genotype: 0.1145  Interaction: 0.8656 | *  ns  ns |
| 5B – Spine density WT  E5 | Two-way ANOVA  Two-way ANOVA | Interaction: p=0.0886  Hairpin: p=0.4370  Drug: p=0.0260  Interaction: p=0.9530  Hairpin: p=0.0685  Drug: p=0.4835 | ns  ns  *  ns  ns  ns |

**Supplementary Table 3: Post-hoc tests and p values for indicated figures.**

| Figure | Post-Hoc Test | P value | Significance |
| --- | --- | --- | --- |
| 1C - NPP | Tukey’s multiple comparisons | WT  S1-S2: p=0.8384  S1-M: p=0.0002  S2-M: p=0.0018  AS  S1-S2: p=0.6892  S1-M: p=0.8469  S2-M: p=0.3582  AS/E5  S1-S2: p=0.0710  S1-M: p<0.0001  S2-M : p=0.0013  E5  S1-S2: p=0.5312  S1-M: p=0.1593  S2-M: p=0.0115 | ns  *  *  ns  ns  ns  ns  *  *  ns  ns  * |
| 1D - NOR | Sidak’s multiple comparisons | WT Familiar-Novel: p=0.0390  AS Familiar-Novel: p=0.7018  AS/E5 Familiar-Novel: p=0.0043  E5 Familiar-Novel: p<0.0001 | *  ns  *  * |
| 1E – Rotarod | Tukey’s multiple comparisons | WT-AS: p=0.0002  WT-AS/E5: p=0.0765  WT-E5: p=0.3876  AS-AS/E5: p=0.6335  AS-E5: p<0.0001  AS/E5-E5: p=0.0074 | *  ns  ns  ns  *  * |
| 1F - Rotarod | Tukey’s multiple comparisons | WT-AS: p=0.0113  WT-AS/E5: p=0.0061  WT-E5: p=0.9969  AS-AS/E5: p=0.9980  AS-E5: p=0.0247  AS/E5-E5: p=0.0142 | *  *  ns  ns  *  * |
| 1G – Marble burying | Tukey’s multiple comparisons | WT-AS: p=0.0010  WT-AS/E5: p=0.0144  WT-E5: p>0.9999  AS-AS/E5: p=0.7471  AS-E5: p=0.0014  AS/E5-E5: p=0.0181 | *  *  ns  ns  *  * |
| 3C – Spine density | Tukey’s multiple comparisons | WT-AS: p=0.0016  WT-AS/E5: p=0.2138  WT-E5: p=0.0002  AS-AS/E5: p<0.0001  AS-E5: p=0.8485  AS/E5-E5: p<0.0001 | *  ns  *  *  ns  * |
| 4B – mEPSC frequency | Tukey’s multiple comparisons | WT-AS: p=0.0020  WT-AS/E5: p=0.8571  WT-E5: p=0.0343  AS-AS/E5: p=0.0120  AS-E5: p=0.8650  AS/E5-E5: p=0.1441 | *  ns  *  *  ns  ns |
| 4E – Rise time | Tukey’s multiple comparisons | WT-AS: p=0.7098  WT-AS/E5: p=0.3257  WT-E5: p=0.1110  AS-AS/E5: p=0.0251  AS-E5: p=0.4956  AS/E5-E5: p=0.0011 | ns  ns  ns  *  ns  * |
| 4F – Decay time | Tukey’s multiple comparisons | WT-AS: p=0.7778  WT-AS/E5: p=0.7826  WT-E5: p=0.2007  AS-AS/E5: p=0.2028  AS-E5: p=0.6243  AS/E5-E5: p=0.0224 | ns  ns  ns  ns  ns  * |
| 1-1E – Passive Avoidance | Bonferroni’s multiple comparisons | Day 1 WT-Day 1 AS: p>0.9999  Day 1 WT-Day 1 AS/E5: p>0.9999  Day 1 WT-Day 1 E5: p>0.9999  Day 1 WT-Day 2 WT: p<0.0001  Day 1 WT-Day 2 AS: p=0.4900  Day 1 WT-Day 2 AS/E5: p>0.9999  Day 1 WT-Day 2 E5: p<0.0001  Day 1 AS-Day 1 AS/E5: p>0.9999  Day 1 AS-Day 1 E5: p>0.9999  Day 1 AS-Day 2 WT: p<0.0001  Day 1 AS-Day 2 AS: p=0.4851  Day 1 AS-Day 2 AS/E5: p>0.9999  Day 1 AS-Day 2 E5: p<0.0001  Day 1 AS/E5-Day 1 E5: p>0.9999  Day 1 AS/E5-Day 2 WT: p<0.0001  Day 1 AS/E5-Day 2 AS: p=0.2648  Day 1 AS/E5-Day 2 AS/E5: p>0.9999  Day 1 AS/E5-Day 2 E5: p<0.0001  Day 1 E5-Day 2 WT: p<0.0001  Day 1 E5-Day 2 AS: p=0.2502  Day 1 E5-Day 2 AS/E5: p=0.9604  Day 1 E5-Day 2 E5: p<0.0001  Day 2 WT-Day 2 AS: p=0.0036  Day 2 WT-Day 2 AS/E5: p=0.0003  Day 2 WT-Day 2 E5: p>0.9999  Day 2 AS-Day 2 AS/E5: p>0.9999  Day 2 AS-Day 2 E5: p=0.0396  Day 2 AS/E5-Day 2 E5: p=0.0035 | ns  ns  ns  *  ns  ns  *  ns  ns  *  ns  ns  *  ns  *  ns  ns  *  *  ns  ns  *  *  *  ns  ns  *  * |
| 1-1F – Passive Avoidance | Bonferroni’s multiple comparisons | Day 1 WT-Day 1 AS: p>0.9999  Day 1 WT-Day 1 AS/E5: p>0.9999  Day 1 WT-Day 1 E5: p>0.9999  Day 1 WT-Day 2 WT: p<0.0001  Day 1 WT-Day 2 AS: p<0.0001  Day 1 WT-Day 2 AS/E5: p<0.0001  Day 1 WT-Day 2 E5: p<0.0001  Day 1 AS-Day 1 AS/E5: p>0.9999  Day 1 AS-Day 1 E5: p>0.9999  Day 1 AS-Day 2 WT: p=0.0004  Day 1 AS-Day 2 AS: p=0.0008  Day 1 AS-Day 2 AS/E5: p=0.0008  Day 1 AS-Day 2 E5: p=0.0008  Day 1 AS/E5-Day 1 E5: p>0.9999  Day 1 AS/E5-Day 2 WT: p<0.0001  Day 1 AS/E5-Day 2 AS: p<0.0001  Day 1 AS/E5-Day 2 AS/E5: p<0.0001  Day 1 AS/E5-Day 2 E5: p<0.0001  Day 1 E5-Day 2 WT: p<0.0001  Day 1 E5-Day 2 AS: p<0.0001  Day 1 E5-Day 2 AS/E5: p<0.0001  Day 1 E5-Day 2 E5: p<0.0001  Day 2 WT-Day 2 AS: p>0.9999  Day 2 WT-Day 2 AS/E5: p>0.9999  Day 2 WT-Day 2 E5: p>0.9999  Day 2 AS-Day 2 AS/E5: p>0.9999  Day 2 AS-Day 2 E5: p>0.9999  Day 2 AS/E5-Day 2 E5: p>0.9999 | ns  ns  ns  *  *  *  *  ns  ns  *  *  *  *  ns  *  *  *  *  *  *  *  *  ns  ns  ns  ns  ns  ns |
| 3-1B – Spine Density | Tukey’s multiple comparisons | WT-AS: p=0.0100  WT-AS/E5: p=0.0601  WT-E5: p<0.0001  AS-AS/E5: p<0.0001  AS-E5: p=0.0086  AS/E5-E5: p<0.0001 | *  ns  *  *  *  * |
| 3-1D – Spine Density | Tukey’s multiple comparisons | WT-AS: p<0.0001  WT-AS/E5: p=0.9997  WT-E5: p<0.0001  AS-AS/E5: p<0.0001  AS-E5: p=0.8737  AS/E5-E5: p<0.0001 | *  ns  *  *  ns  * |
| 3-1F – Spine Density | Tukey’s multiple comparisons | WT-AS: p=0.0033  WT-AS/E5: p=0.0074  WT-E5: p=0.0276  AS-AS/E5: p<0.0001  AS-E5: p=0.8394  AS/E5-E5: p<0.0001 | *  *  *  *  ns  * |
| 4-1C – Input/Output | Sidak’s multiple comparisons | WT-AS Input 20: p>0.9999  WT-AS Input 30: p>0.9999  WT-AS Input 40: p>0.9999  WT-AS Input 50: p>0.9999  WT-AS Input 75: p=0.9956  WT-AS Input 100: p=0.8323  WT-AS Input 200: p=0.0182  WT-AS Input 300: p=0.0135  WT-AS Input 400: p=0.0175  WT-AS Input 500: p=0.0261 | ns  ns  ns  ns  ns  ns  *  *  *  * |
| 4-1D – PPF | Sidak’s multiple comparisons | WT-AS ISI: p=0.9992  WT-AS ISI: p=0.3286  WT-AS ISI: p>0.9999  WT-AS ISI: p=0.9437  WT-AS ISI: p=0.9121 | ns  ns  ns  ns  ns |
| 4-1F – Input/Output | Sidak’s multiple comparisons | WT-AS/E5^-/-^ Input 20: p>0.9999  WT-AS/E5^-/-^ Input 30: p>0.9999  WT-AS/E5^-/-^ Input 40: p>0.9999  WT-AS/E5^-/-^ Input 50: p>0.9999  WT-AS/E5^-/-^ Input 75: p=0.8277  WT-AS/E5^-/-^ Input 100: p=0.7220  WT-AS/E5^-/-^ Input 200: p=0.0638  WT-AS/E5^-/-^ Input 300: p=0.0676  WT-AS/E5^-/-^ Input 400: p=0.1010  WT-AS/E5^-/-^ Input 500: p=0.0069 | ns  ns  ns  ns  ns  ns  ns  ns  ns  * |
| 4-1H – PPF | Sidak’s multiple comparisons | WT-AS/E5^-/-^ ISI: p=0.6615  WT-AS/E5^-/-^ ISI: p=0.7465  WT-AS/E5^-/-^ ISI: p=0.9605  WT-AS/E5^-/-^ ISI: p>0.9999  WT-AS/E5^-/-^ ISI: p=0.9959 | ns  ns  ns  ns  ns |
| 5B – Spine density WT  E5 | Sidak’s multiple comparisons  Sidak’s multiple comparisons | WT Scr DMSO-WT Scr CNO: p=0.9988  WT Scr DMSO-WT Ub DMSO: p=9849  WT Scr DMSO- WT Ub CNO: p=0.1766  WT Scr CNO- WT Ub DMSO: p=0.8670  WT Scr CNO- WT Ub CNO: p=0.3815  WT Ub DMSO- WT Ub CNO: p=0.0468  E5 Scr DMSO-E5 Scr CNO: p=0.9952  E5 Scr DMSO-E5 Ub DMSO: p=7705  E5 Scr DMSO- E5 Ub CNO: p=0.9540  E5 Scr CNO- E5 Ub DMSO: p=0.4045  E5 Scr CNO- E5 Ub CNO: p=0.6691  E5 Ub DMSO- E5 Ub CNO: p=0.9982 | ns  ns  ns  ns  ns  *  ns  ns  ns  ns  ns  ns |

Armbruster, B.N., Li, X., Pausch, M.H., Herlitze, S., and Roth, B.L. (2007). Evolving the lock to fit the key to create a family of G protein-coupled receptors potently activated by an inert ligand. Proc Natl Acad Sci U S A *104*, 5163-5168.

Greer, P.L., Hanayama, R., Bloodgood, B.L., Mardinly, A.R., Lipton, D.M., Flavell, S.W., Kim, T.K., Griffith, E.C., Waldon, Z., Maehr, R.*, et al.* (2010). The Angelman Syndrome protein Ube3A regulates synapse development by ubiquitinating arc. Cell *140*, 704-716.

Judson, M.C., Wallace, M.L., Sidorov, M.S., Burette, A.C., Gu, B., van Woerden, G.M., King, I.F., Han, J.E., Zylka, M.J., Elgersma, Y.*, et al.* (2016). GABAergic Neuron-Specific Loss of Ube3a Causes Angelman Syndrome-Like EEG Abnormalities and Enhances Seizure Susceptibility. Neuron *90*, 56-69.

Margolis, S.S., Salogiannis, J., Lipton, D.M., Mandel-Brehm, C., Wills, Z.P., Mardinly, A.R., Hu, L., Greer, P.L., Bikoff, J.B., Ho, H.Y.*, et al.* (2010). EphB-mediated degradation of the RhoA GEF Ephexin5 relieves a developmental brake on excitatory synapse formation. Cell *143*, 442-455.

Rodriguez, A., Ehlenberger, D.B., Dickstein, D.L., Hof, P.R., and Wearne, S.L. (2008). Automated three-dimensional detection and shape classification of dendritic spines from fluorescence microscopy images. PLoS One *3*, e1997.
